# Beyond point estimates. A comment on: ‘The environmental cost of welfare-driven policy changes in UK egg production’ (2026), by *Martinic et al*.

**DOI:** 10.1098/rsos.261749

**Published:** 2026-11-11

**Authors:** Ilias Kyriazakis

**Affiliations:** School of Biological Sciences, Queen's University Belfast, Belfast, UK

**Keywords:** animal welfare, environmental impact, egg, life cycle assessment, laying hen, uncertainty

## Abstract

A recent study comparing UK egg production systems concluded that the current (‘business-as-usual’) UK egg production system consisting of a mix of cage and non-cage systems minimizes environmental impacts, requires fewer laying hens and results in lower mortality than alternative cage-free scenarios. While the objective of evaluating environmental and welfare consequences of production system changes is important, I argue that these conclusions are not sufficiently supported by the methodology employed. The analysis relies on life cycle inventory and mortality data generated around 2010 to represent the UK industry in 2022, despite substantial advances in genetics, nutrition, housing, flock health and management over the intervening period. More importantly, it disregards the uncertainty associated with the original environmental and production datasets, treating inherently variable biological systems as deterministic. This omission is compounded by scaling farm-level point estimates to national production, thereby magnifying apparent differences between scenarios without quantifying their statistical significance. I contend that robust policy recommendations regarding transitions between egg production systems require analyses based on contemporary production data, explicit propagation of uncertainty and integrated evaluation of environmental sustainability and animal welfare.

*R. Soc. Open Sci.* **13**, 251830. (Published online 29 July 2026). (https://doi.org/10.1098/rsos.251830)

## 1. Introduction

In a recent paper published in *Royal Society Open Science* [1], the authors compare alternative laying-hen production systems in the United Kingdom with respect to their environmental impacts and welfare consequences. Welfare is represented using laying-hen mortality as an ‘iceberg’ indicator, while environmental impacts are estimated through life cycle assessment (LCA). They conclude that the current (‘business-as-usual’) UK egg production system, comprising a mix of cage and non-cage systems, results in lower environmental impacts, requires fewer laying hens and produces lower mortality than alternative cage-free scenarios.

Evaluating the consequences of changes in national egg production systems is both timely and important. However, I suggest that the conclusions drawn by the authors are not sufficiently supported by the methodology employed. Their analysis relies heavily on historical production data, omits uncertainty associated with the underlying life cycle inventory and extrapolates farm-level estimates to national production without considering how uncertainty propagates through this scaling process.

## 2. The omission of uncertainty in the environmental assessment

The environmental inventory underpinning the analysis is derived principally from the studies of Leinonen *et al.* [2,3]. These datasets were generated from measurements and production records collected around 2010 as part of a comprehensive UK-wide research programme designed to provide statistically robust comparisons of laying-hen production systems.

A major strength of the original work was its explicit treatment of uncertainty. Rather than reporting single deterministic estimates, Leinonen *et al.* [2,3] incorporated measurement error, biological variation, variability in production parameters and uncertainty in inventory data throughout the LCA. Environmental impacts were therefore presented with confidence intervals, allowing formal statistical comparisons between systems.

This distinction is important because the original analyses showed that, although conventional cage systems generally produced lower point estimates for several environmental indicators, many apparent differences between production systems were not statistically distinguishable once uncertainty was considered. Their conclusions were therefore appropriately qualified by the uncertainty surrounding the estimates.

By contrast, the present study adopts the point estimates from Leinonen *et al.* [2,3] but omits their associated uncertainty. Environmental impacts are consequently treated as deterministic values despite being derived from inherently variable biological production systems. Relatively small numerical differences are therefore implicitly interpreted as meaningful even though the original studies indicate that many may not be statistically significant.

The consequences become more pronounced when these deterministic values are scaled to national production. Small differences at farm level become magnified into apparently substantial differences when multiplied by national egg production volumes. Had uncertainty been propagated through these calculations, the resulting confidence intervals around national estimates would almost certainly overlap to a much greater extent. Mathematical scaling does not increase confidence in the estimates; it amplifies the consequences of uncertainty that has not been quantified.

## 3. Methodological limitations

There are also methodological differences between the present study and the original environmental assessments that may influence the reported environmental impacts. Although both studies estimate lower point values for cage systems than for non-cage systems, the absolute impacts reported by [1] are systematically lower than those presented by Leinonen *et al.* [2,3], suggesting that differences arise from changes in life cycle inventory assumptions rather than improvements in production efficiency alone.

One example concerns feed production. Leinonen *et al.* [2,3] estimated feed-related impacts using methodologies developed within the UK national inventory framework described by Williams *et al.* [4], whereas [1] derives feed ingredient impacts from the ECOALIM database, which is based primarily on French agricultural data. Similarly, relatively little methodological detail is provided for manure management and associated emissions. The original analyses relied on Defra-funded research available at the time [4], but emission factors have subsequently been revised for several laying-hen manure management pathways. These differences may influence estimates of ammonia emissions and the associated acidification and eutrophication potentials, thereby increasing the apparent environmental advantage of furnished cages.

The study also relies on environmental inventories and production parameters originating largely from around 2010 to represent the UK laying industry in 2022. During this period, substantial advances have occurred in genetics, nutrition, housing, flock health, biosecurity and productivity, and these improvements have not necessarily occurred uniformly across production systems. Consequently, there is little justification for assuming that environmental performance measured more than a decade earlier accurately represents contemporary production.

The welfare assessment is subject to a similar concern because it considers only hen mortality as the welfare indicator and adopts mortality values directly from the historical datasets. This approach assumes that hen mortality rates have remained unchanged, despite well-documented improvements in genetics, health management, housing design and husbandry practices [5]. These advances have been particularly influential in free-range and organic systems, where mortality has declined with increasing management experience and the development of genotypes better suited to non-cage environments [5]. As a result, the application of historical mortality estimates to present-day production systems is likely to overestimate mortality and, in turn, exaggerate the laying-hen numbers required under alternative production scenarios.

Because estimates of national laying-hen populations depend directly on historical productivity and mortality parameters, they should also be interpreted cautiously until uncertainty surrounding these parameters has been propagated through the analysis.

## 4. Conclusion

The authors conclude that future animal welfare policy should account for the environmental impacts of production before major legislative changes are introduced. I fully support this principle. However, policy recommendations must rest on robust and transparent analyses that appropriately account for the uncertainty inherent in biological production systems and are based on data that accurately reflect contemporary production practices.

Methodological frameworks are now available that explicitly integrate animal welfare and environmental sustainability within a common decision-making framework while quantifying uncertainty and trade-offs between competing objectives (e.g. [6,7]). Such approaches provide a more rigorous basis for informing policy than deterministic comparisons based on historical datasets.

Irrespective of housing system, improving animal performance, health and longevity while reducing mortality should remain central objectives of sustainable egg production. Any transition towards non-cage systems should therefore be evaluated not only in terms of environmental impacts and the number of laying hens required to meet production targets, but also with respect to the quality of life experienced by those animals [8]. Likewise, continued improvements within existing production systems may deliver substantial gains in both environmental performance and animal welfare.

## Data Availability

This article has no additional data.
